# Trauma-induced complete pacemaker lead fracture 8 months prior to hospitalization: A case report

**DOI:** 10.1515/med-2021-0327

**Published:** 2021-10-04

**Authors:** Yi Xu, Xiaoming Chen, Jianyi Feng, Jun Guo, Zicheng Li

**Affiliations:** Department of Cardiology, First Affiliated Hospital of Ji’Nan University, No. 613 Huangpu Avenue West, Tianhe District, Guangzhou, 510630, China

**Keywords:** pacemaker, lead fracture, complication

## Abstract

Trauma-induced complete lead fracture is a rare complication of pacemaker implantation. Only a few cases have been previously reported. Common treatment included replacement of pacemaker and/or extraction of fractured lead. In this report, however, we describe this unique case of complete traumatic pacemaker lead fracture. The patient had her right-ventricular lead fractured after a bicycle accident and had lived with the fractured lead for 8 months prior to her hospitalization. After examinations, she was treated with a relatively conservative strategy. The pacemaker and fractured lead were left for further observation and follow-up.

## 1 Introduction

Despite its extremely low incidence, trauma-induced lead fracture remains a feared complication of pacemaker implantation [1,2]. In patients who are pacemaker-dependent, this event can be life-threatening [1]. A few cases have been reported and these patients were all treated within days with pacemaker replaced and/or fractured lead removed [2,3,4]. Here, we present this unique case of a complete traumatic pacemaker lead fracture. The patient had lived with a fractured lead for 8 months prior to hospitalization and was treated with a relatively conservative strategy.

## 2 Case presentation

**Figure 1:**
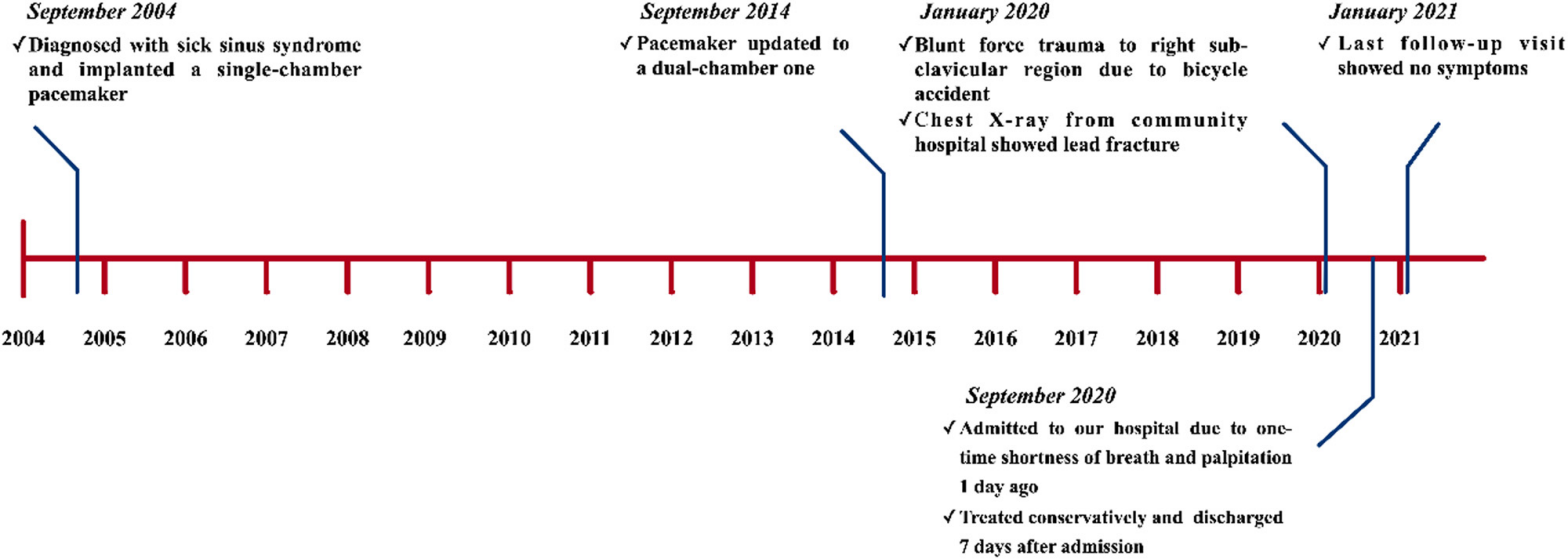
Case timeline.

**Figure 2:**
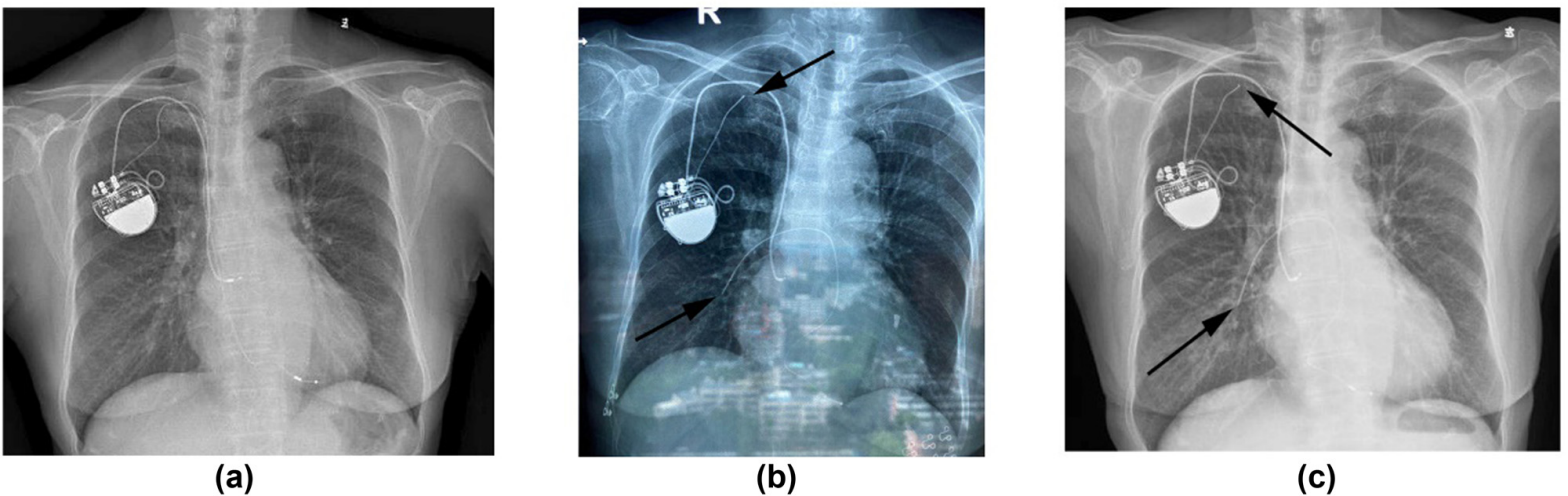
Chest X-ray images from December 2019 (a) showing both leads were intact, while results from January 2020 (b) and September 2020 (c) showing a complete right-ventricular lead fracture (arrow).

A 71-year-old woman visited our hospital on September 2, 2020 due to shortness of breath and palpitation after exercise 1 day ago. The symptoms occurred once only and were inactive on admission. Figure 1 shows the timeline of this case. She was diagnosed with sick sinus syndrome with normal atrioventricular conduction and had a single-chamber AAI pacemaker implanted in 2004. The pacemaker was implanted on the right side because of left-handedness. The battery ran out 10 years later and she was admitted to our hospital in 2014. Similar single-chamber pacemakers were not available and not covered by the patient’s medical care program anymore. Moreover, her atrioventricular node function had shown to be slightly reduced with age. Thus, the pacemaker was upgraded to a dual-chamber one back then. The atrial lead was kept, while a new ventricular lead was implanted. The patient did not routinely go to follow-up visits in the following 5 years. A chest X-ray image from December 2019 showed that both leads were intact (Figure 2a). Eight months prior to this admission (January 2020), she fell off from a running bicycle, causing blunt force trauma to her right subclavicular region. She went to a community hospital and underwent a chest X-ray screening. It showed a complete right-ventricular pacemaker lead fracture (Figure 2b). The fractured lead was seen at the level of the right clavicle, while the proximal end of the lead was still attached to the pacemaker. The atrial lead was still intact. The patient was suggested to go to a superior hospital for further evaluation and treatment. However, she refused to do so, thinking that it was unnecessary due to the lack of any specific symptoms back then. She was then discharged and did not have any medical visits or hospitalization until this time. Her comorbidities included stable angina and hypertension.

**Figure 3:**
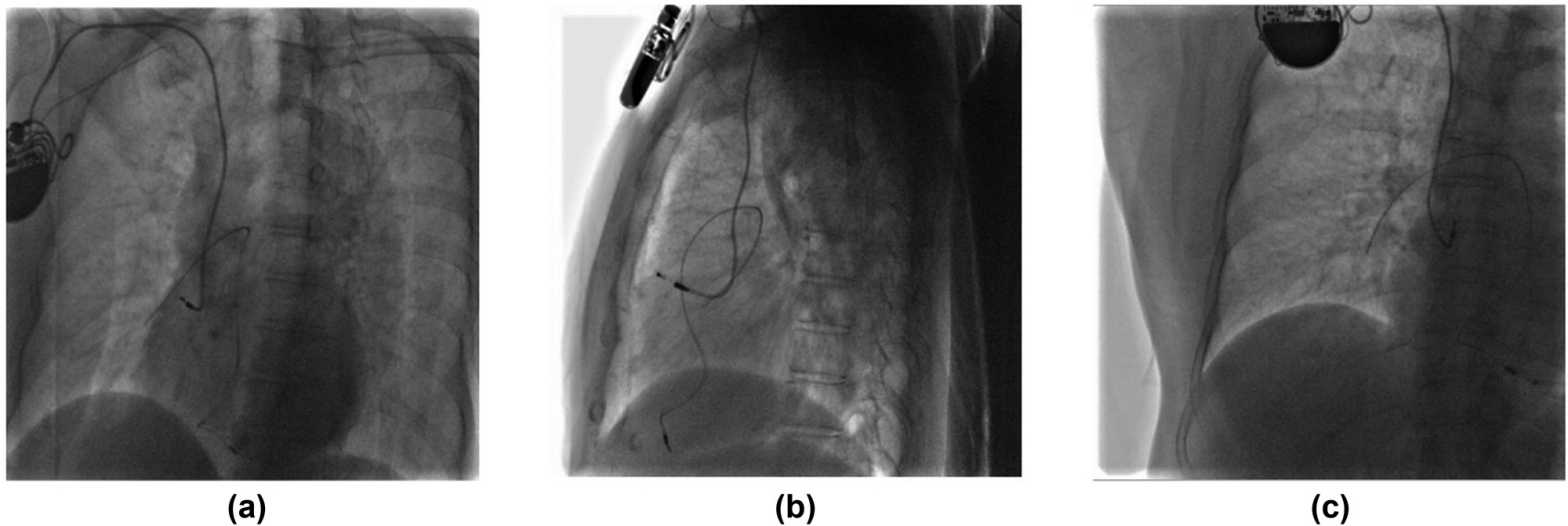
Chest fluoroscopy from different body positions (a–c).

Physical and laboratory examinations on this admission showed no significant findings. Myocardial enzyme test had negative results. Her electrocardiography (ECG) showed atrial pacemaker rhythm and no specific ST-T changes. Chest X-ray scanning had similar findings as compared with 8 months ago, showing a complete ventricular lead fracture (Figure 2c). The results of chest fluoroscopy confirmed that the free end of the fractured ventricular lead was in a fixed position and did not move with heartbeats or change of body position (Figure 3). The patient then underwent a 24 h Holter monitor to further examine the status of her pacemaker. The results showed satisfying atrial sensing and pacing with an AAI pacing mode. Further examinations including coronary angiography and coronary CT angiography were refused by the patient. After symptomatic treatment and observation, the patient was discharged 7 days after admission. The patient had paid follow-up visits routinely after discharge and there were no symptoms up to her latest visit on January 12, 2021.

**Ethics statement:** The patient has provided informed consent for the publication of this study and for the use of her medical data.

## 3 Discussion

Pacemakers play an important role in the treatment and management of patients with bradyarrhythmias. Yet, complications after implantation can lead to significantly worse outcomes [5]. Leads are considered the most vulnerable part of the pacemaker device. The reported incidence rate of lead fracture ranges from 0.1 to 4.2% per year [1,5].

Crush injury, also known as subclavian crush syndrome, is a delayed complication of pacemaker implantation that can also cause lead fracture [6,7]. Lead fractures caused by it [5] are more frequently seen and reported than those caused by trauma or injury [2,3,4]. However, it was not considered the reason for lead fracture in this case. Although it is not possible to monitor the patient 24/7, a chest X-ray image from December 2019 showed that both leads were intact at least a month before the accident (Figure 2a). Damage from subclavian crush syndrome is a gradual process, it is unlikely that it could cause such complete fracture in a short time period. Moreover, crush injury should do damage to both leads, while the atrial lead was still intact and working properly here. Therefore, impact from blunt trauma was considered the main cause of ventricular lead fracture. It is possible that when the ventricular lead was implanted in 2014, the puncture site was apart from the older atrial lead. This made the ventricular lead closer to the body surface and more vulnerable to impact.

Therefore, this rare etiology should be considered by physicians whenever the pacemaker-implanted patients had a history of injury or trauma to the chest, especially the subclavicular region. Considering the anatomy of the subclavian vein, pacemakers implanted at the right subclavicular region are more likely to fracturing after force or trauma [1]. ECG may help detect lead fracture and dysfunction. Chest X-ray is the most direct way to diagnose a lead fracture and determine the status of the fractured lead. Chest fluoroscopy, though not frequently performed nowadays, is helpful to evaluate the fractured lead’s movement with heartbeats and body position changes. A 24 h Holter monitor is considered necessary in evaluating the status of the pacemaker. The results of these examinations will be helpful for decision- and strategy-making regarding further treatment in these cases.

What distinguished this case from previously reported ones is that she had lived with a fractured ventricular lead for a long time before she was admitted and managed in a relatively conservative way. All previously reported patients received treatment within days after lead fracture [2,3,4]. Common treatment strategies for trauma-induced complete pacemaker lead fracture include replacement of a new pacemaker and/or removal of the fractured lead, as previously described [2,3,4,8]. However, in this case, the patient was managed in a conservative manner. Her right-ventricular lead was already fractured 8 months prior to her admission in September 2020. This means her dual-chamber pacemaker had been working with a single atrial lead and an AAI mode since then. The sensing and pacing function of her pacemaker was still satisfying, which might explain the reason she had no symptoms relevant to the pacemaker lead to fracture, and did not seek any medical treatment for the next 8 months. As for the transient symptoms the day before her hospitalization, it is possible that what she had was actually another onset of angina, though further examinations were refused. No symptoms occurred after discharge. Thus, it is acceptable to leave her pacemaker for further observation and follow-up.

Moreover, fractured lead removal was considered a challenging process and may be complicated by vascular rupture, pneumothorax, cardiac perforation, tamponade, or even death [9]. In this case, chest fluoroscopy indicated that the free end of the fractured lead was in a fixed position. It is possible that it was fixed to vessels like the pulmonary artery. Removing it might risk causing severe complications including pulmonary artery rupture or embolism. Therefore, we did not perform any extractions.

The key learning point from this case is that when encountering patients with trauma-induced complete pacemaker lead fracture, the function of the pacemaker and risks of lead extraction should be evaluated thoroughly before decision-making. If the pacemaker still has satisfying function and the patient does not have directly relevant symptoms, then it is acceptable to manage the patient in a more conservative way.
